# P-1135. Genomic diversity of global colonizing multidrug-resistant Escherichia coli isolates across multiple high prevalence resource-limited settings

**DOI:** 10.1093/ofid/ofaf695.1329

**Published:** 2026-01-11

**Authors:** Ahmed Babiker, Rafael Araos, Fahmida Chowdhury, Lorena Díaz, Mary K Hayden, Ebbing Lautenbach, Naledi Mannathoko, Jose R W Martínez, Mosepele Mosepele, Jose M Munita, Nure Sharaf Nower Samia, Dhatri Badri Narayanan, Evan Snitkin

**Affiliations:** Rush University Medical Center, Chicago, IL; Instituto de Ciencias e Innovación en Medicina, Facultad de Medicina Clínica Alemana Universidad del Desarrollo, Santiago, Region Metropolitana, Chile; icddr,b, Dhaka, Dhaka, Bangladesh; Universidad del Desarrollo - Clinica Alemana, Santiago, Region Metropolitana, Chile; Rush University Medical Center, Chicago, IL; University of Pennsylvania, Philadelphia, Pennsylvania; University of Botswana, Philadelphia, Pennsylvania; Genomics & Resistant Microbes (GeRM), Instituto de Ciencias e Innovación en Medicina, Facultad de Medicina Clínica Alemana, Universidad del Desarrollo, Chile; Millennium Initiative for Collaborative Research on Bacterial Resistance (MICROB-R), Santiago, Region Metropolitana, Chile; University of Botswana, Philadelphia, Pennsylvania; Facultad de Medicina Clinica Alemana - Universidad del Desarrollo, Santiago, Region Metropolitana, Chile; International Centre for Diarrheal Disease Research, Bangladesh, Dhaka, Dhaka, Bangladesh; University of Michigan, Ann Arbor, Michigan; University of Michigan, Ann Arbor, Michigan

## Abstract

**Background:**

Antimicrobial resistance (AMR) is a major global health threat, with the greatest burden observed in resource-limited settings. Prior work by the Antibiotic Resistance in Communities and Hospitals (ARCH) research consortium revealed a high burden of multidrug-resistant *Escherichia coli* (MDRE) colonization in both community and healthcare settings in 6 low- and middle-income countries. We aimed to characterize the genomic diversity of MDRE colonizing strains recovered in ARCH studies to provide a high-resolution picture of AMR in community and healthcare networks on a global scale.

**Table 1.**
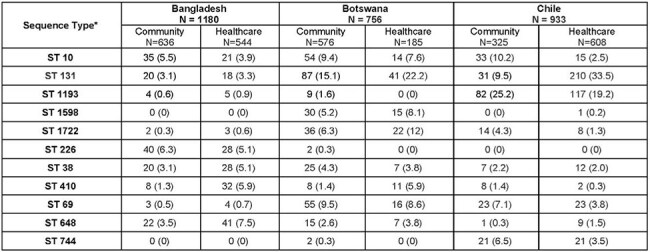
Distribution of most frequent major colonizing multidrug-resistant Escherichia coli sequence types across community and healthcare settings recovered from the Bangladesh, Botswana and Chile Antibiotic Resistance in Communities and Hospitals study sites (N=2874) *The five most frequently observed sequence types from each site were included

**Figure 1.**
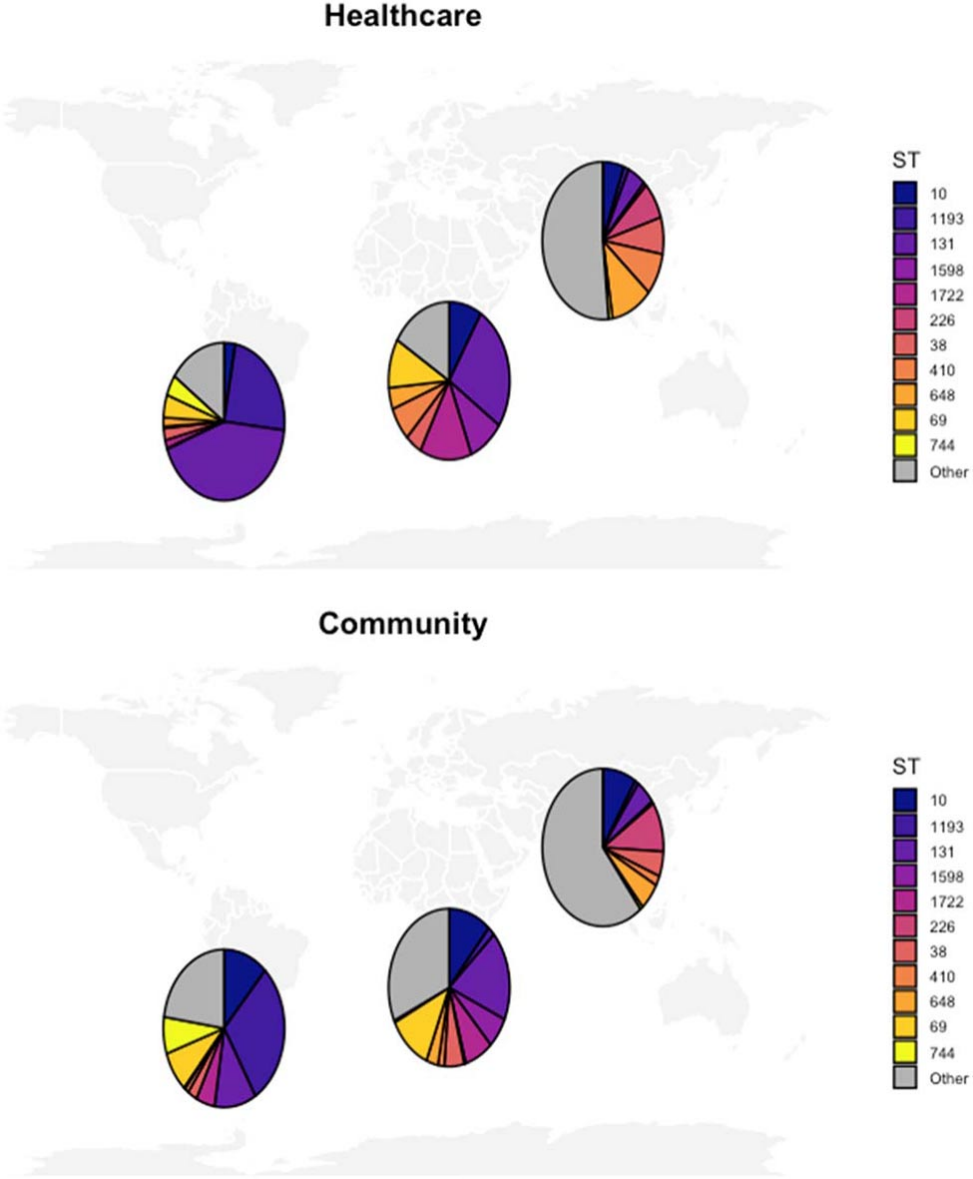
Distribution of most frequent major colonizing multidrug-resistant Escherichia coli sequence types across community and healthcare settings recovered from the Bangladesh, Botswana and Chile Antibiotic Resistance in Communities and Hospitals study sites For data visualization purposes, the five most frequently observed sequence types from each site were included.

**Methods:**

A cross-sectional, population-based prevalence survey was conducted between December 2018 and March 2022. Fecal and perirectal specimens were collected from hospitalized patients and community participants and plated to selective agars. Target phenotypes included extended-spectrum cephalosporin-resistant (all sites), carbapenem-resistant (all sites) and fluoroquinolone-resistant *E. coli* (Chile) confirmed through susceptibility testing. MDRE isolates underwent whole genome sequencing (WGS). WGS data from three sites (Bangladesh, Botswana and Chile) underwent quality check (QC), trimming, assembly, species and sequence type (ST) calling. *E. coli* STs with ≥10 isolates were classified as major STs and those with < 10 isolates were classified as minor.

**Figure 2.**
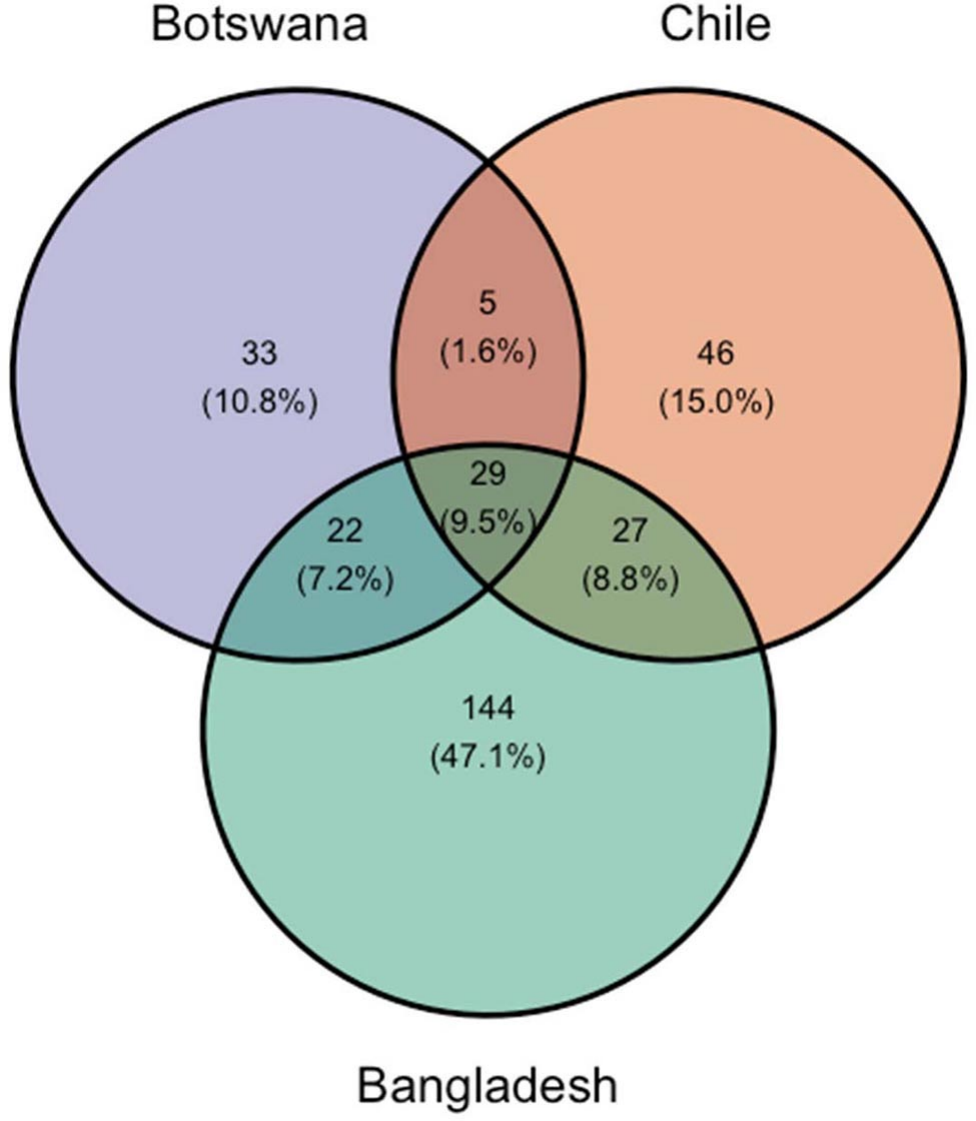
Venn Diagram of unique and shared colonizing multidrug-resistant Escherichia coli sequence types recovered from the Bangladesh, Botswana and Chile Antibiotic Resistance in Communities and Hospitals study sites

**Results:**

A total of 2874 (Bangladesh:1180, Botswana:756, Chile:933) MDRE isolates passed QC and were included. Among recovered MDRE isolates, 306 unique STs (42 major, 264 minor) were observed, 222 in Bangladesh, 107 in Chile and 89 in Botswana. Overall, the five most frequent STs were ST131 (N=407), ST1193 (N=217), ST10 (N=172), ST69 (N=124) and ST 38 (N=99)(Table 1, Figure 1). Only 29 (20 major, 9 minor) STs were shared across all three site (Figure 2). Across healthcare and community settings 127 STs were shared, 73 STs were detected exclusively in the healthcare setting and 106 ST were detected exclusively in the community setting.

**Conclusion:**

Colonizing MDRE strains from global low-resource settings exhibited considerable diversity across disparate global sites with ST overlap between community and healthcare settings.

**Disclosures:**

Ahmed Babiker, MBBS, MSc, Beckman Coulter Inc.: Advisor/Consultant Jose M. Munita, MD, MSD: Grant/Research Support|Pfizer: Grant/Research Support

